# Supplementary material for: Coupling of mitochondrial function and skeletal muscle fiber type by a miR‐499/Fnip1/AMPK circuit
Source: EMBO Mol Med. 2016 Aug 9;8(10):1212–28. doi: 10.15252/emmm.201606372 (PMC5048369; doi:10.15252/emmm.201606372)
Supplement: Supplementary file 1 — Appendix [file EMMM-8-1212-s001.pdf]

Appendix

Coupling of mitochondrial function and skeletal muscle fiber type by a miR-499/Fnip1/AMPK circuit

Jing Liu[1,4], Xijun Liang[1,4], Danxia Zhou[1], Ling Lai[2], Liwei Xiao[1], Lin Liu[1], Tingting Fu[1], Yan Kong[3], Qian Zhou[1], Rick B. Vega[2], Min-Sheng Zhu[1], Daniel P. Kelly[2], Xiang Gao[1] and Zhenji Gan[1,5]

Table of Contents

Appendix Table S1
Appendix Table S2
Appendix Table S3
Appendix Table S4

**Appendix Table S1. List of calcium signaling and muscle contraction genes up-regulated in MCK-miR-499 gastrocnemius muscle**		
**Gene**	**Description**	**Fold Change**
Ablim1	actin-binding LIM protein 1	1.5
Actn2	actinin alpha 2	1.5
Arpc1b	actin related protein 2/3 complex, subunit 1B	1.4
Atp2a2	ATPase, Ca++ transporting, cardiac muscle, slow twitch 2	2.6
Casq2	calsequestrin 2 (cardiac muscle)	1.8
Chrna1	cholinergic receptor, nicotinic, alpha 1 (muscle)	4.6
Chrnd	cholinergic receptor, nicotinic, delta (muscle)	1.3
Csrp3	cysteine and glycine-rich protein 3	2.3
Dmd	dystrophin, muscular dystrophy	1.9
Enah	enabled homolog (Drosophila)	1.6
Gys2	glycogen synthase 2	1.5
Hdac9	histone deacetylase 9	1.8
Micu1	mitochondrial calcium uptake 1	1.4
Murc	muscle-related coiled-coil protein	1.3
Myh1	myosin, heavy polypeptide 2, skeletal muscle, adult; myosin, heavy polypeptide 1, skeletal muscle, adult	1.5
Myh2	myosin, heavy chain 2, skeletal muscle, adult	1.3
Myh6	myosin, heavy polypeptide 6, cardiac muscle, alpha	1.9
Myh7	myosin, heavy polypeptide 7, cardiac muscle, beta	3.2
Myh8	myosin, heavy polypeptide 8, skeletal muscle, perinatal	1.5
Myl2	myosin, light polypeptide 2, regulatory, cardiac, slow	2.9
Myl3	myosin, light polypeptide 3	5.5
Myl6b	myosin, light polypeptide 6B	1.6
Myoz2	myozenin 2	3.1
Pgm5	phosphoglucomutase 5	1.7
Ptpn11	protein tyrosine phosphatase, non-receptor type 11	1.4
Rap1a	RAP1A, member of RAS oncogene family	1.6
Rcan2	regulator of calcineurin 2	2.0
Rhou	ras homolog gene family, member U	1.5
Slc8a1	solute carrier family 8 (sodium/calcium exchanger), member 1	2.2
Slc8a3	solute carrier family 8 (sodium/calcium exchanger), member 3	2.2
Slmap	sarcolemma associated protein	1.9
Smtnl1	smoothelin-like 1	3.2
Tnnc1	troponin C type 1 (slow)	7.9
Tnni1	troponin I type 1 (skeletal, slow)	6.7
Tnnt1	troponin T type 1 (skeletal, slow)	9.0
Tnnt2	troponin T2, cardiac	3.1
Tpm3	predicted gene 7848; predicted gene 7839; predicted gene 4157; similar to tropomyosin 3, gamma; tropomyosin 3, gamma; predicted gene 4903	3.1
Trdn	triadin	4.2

Ush1g	Usher syndrome 1G homolog (human)	1.3

Genes involved in calcium signaling and muscle contraction (Gene Ontology) were compared between NTG and MCK-miR-499 gastrocnemius. 39 up-regulated genes (fold change greater than 1.3 and a significant p value < 0.05) are shown.

**Appendix Table S2. List of mitochondrial related genes up-regulated in MCK-miR-499 gastrocnemius muscle**		
**Gene**	**Description**	**Fold Change**
Abcd2	ATP-binding cassette, sub-family D (ALD), member 2	1.6
Acad8	acyl-Coenzyme A dehydrogenase family, member 8	1.4
Acadl	acyl-Coenzyme A dehydrogenase, long-chain	1.6
Acot13	acyl-CoA thioesterase 13	1.3
Acsl1	acyl-CoA synthetase long-chain family member 1	1.4
Agpat5	1-acylglycerol-3-phosphate O-acyltransferase 5 (lysophosphatidic acid acyltransferase, epsilon)	1.4
Agxt2l2	alanine-glyoxylate aminotransferase 2-like 2	1.3
Akr1b10	RIKEN cDNA 2310005E10 gene	1.3
Aldh2	aldehyde dehydrogenase 2, mitochondrial	1.4
Armc1	armadillo repeat containing 1	1.5
Atp5e	ATP synthase, H+ transporting, mitochondrial F1 complex, epsilon subunit	1.6
Atp5g1	predicted gene 12337; predicted gene 10039; predicted gene 4479; ATP synthase, H+ transporting, mitochondrial F0 complex, subunit c (subunit 9), isoform 1	1.3
Atp5l	predicted gene 10221; predicted gene 4602; predicted gene 9391; ATP synthase, H+ transporting, mitochondrial FO complex, subunit G2, pseudogene; predicted gene 11477; predicted gene 9712; ATP synthase, H+ transporting, mitochondrial F0 complex, subunit g; predicted gene 4045	1.5
Bbox1	butyrobetaine (gamma), 2-oxoglutarate dioxygenase 1 (gamma-butyrobetaine hydroxylase)	9.4
Bnip3	predicted gene 14506; BCL2/adenovirus E1B interacting protein 3; predicted gene 6532; similar to E1B 19K/Bcl-2-binding protein homolog	1.4
Brp44	brain protein 44; similar to brain protein 44; predicted gene 3982	1.7
Cabc1	chaperone, ABC1 activity of bc1 complex like (S. pombe)	1.3
Cbara1	calcium binding atopy-related autoantigen 1	1.4
Cox6c	predicted gene 6265; similar to cytochrome c oxidase, subunit VIc; cytochrome c oxidase, subunit VIc	1.8
Cox7a1	cytochrome c oxidase, subunit VIIa 1	1.6
Cox7b	cytochrome c oxidase subunit VIIb	1.5
Crat	carnitine acetyltransferase	1.4
Cycs	predicted gene 10108; cytochrome c, somatic; predicted gene 10053; similar to cytochrome c	1.3
D9Ertd402e	DNA segment, Chr 9, ERATO Doi 402, expressed	1.5
Dci	dodecenoyl-Coenzyme A delta isomerase (3,2 trans-enoyl-Coenyme A isomerase)	1.5
Efha1	EF hand domain family A1	1.4
Etfa	predicted gene 2893; electron transferring flavoprotein, alpha polypeptide	1.3
Fam36a	predicted gene 15683; RIKEN cDNA 2310005N03 gene; similar to RIKEN cDNA 2310005N03	1.4
Gatc	glutamyl-tRNA(Gln) amidotransferase, subunit C homolog (bacterial)	1.5
Gbas	glioblastoma amplified sequence	1.4
Gimap5	GTPase, IMAP family member 5	1.6
Glrx	glutaredoxin	1.4

Gm9108	predicted gene 13910; similar to Hydroxyacyl-Coenzyme A dehydrogenase/3-ketoacyl-Coenzyme A thiolase/enoyl-Coenzyme A hydratase (trifunctional protein), beta subunit; hydroxyacyl-Coenzyme A dehydrogenase/3-ketoacyl-Coenzyme A thiolase/enoyl-Coenzyme A hydratase (trifunctional protein), beta subunit; predicted gene 9108	1.5
Got2	glutamate oxaloacetate transaminase 2, mitochondrial	1.3
Gpx1	glutathione peroxidase 1	1.3
Grsf1	G-rich RNA sequence binding factor 1	1.4
Gtpbp10	GTP-binding protein 10 (putative); predicted gene 6877	1.5
Hccs	holocytochrome c synthetase	1.8
Hint2	histidine triad nucleotide binding protein 2	1.3
Hsdl2	hydroxysteroid dehydrogenase like 2	1.5
Hspb7	heat shock protein family, member 7 (cardiovascular)	1.4
Idh1	isocitrate dehydrogenase 1 (NADP+), soluble	1.3
Idh2	isocitrate dehydrogenase 2 (NADP+), mitochondrial	1.5
Immp1l	IMP1 inner mitochondrial membrane peptidase-like (S. cerevisiae)	1.7
Immp2l	IMP2 inner mitochondrial membrane peptidase-like (S. cerevisiae)	1.6
Isca1	iron-sulfur cluster assembly 1 homolog (S. cerevisiae)	1.5
Ldhb	lactate dehydrogenase B; predicted gene 5514	3.7
Lyrm5	LYR motif containing 5	1.7
Lyrm7	LYR motif containing 7	1.3
Maob	monoamine oxidase B	2.7
Mapk10	mitogen-activated protein kinase 10	1.8
Mavs	mitochondrial antiviral signaling protein	1.3
Mcee	methylmalonyl CoA epimerase	1.5
Mdh1	malate dehydrogenase 1, NAD (soluble)	1.7
Mgst1	microsomal glutathione S-transferase 1	1.7
Mrps15	mitochondrial ribosomal protein S15	1.3
Mrps33	mitochondrial ribosomal protein S33; predicted gene 12540	1.6
Mrps36	predicted gene 10078; predicted gene 3544; similar to mitochondrial ribosomal protein S36; mitochondrial ribosomal protein S36; predicted gene 7258; predicted gene 4676	1.3
Mrrf	mitochondrial ribosome recycling factor; similar to mitochondrial ribosome recycling factor; predicted gene 6374	1.4
Msrb2	methionine sulfoxide reductase B2	1.5
Mtfr1	mitochondrial fission regulator 1	1.3
Ndufa1	NADH dehydrogenase (ubiquinone) 1 alpha subcomplex, 1	1.7
Ndufa4	NADH dehydrogenase (ubiquinone) 1 alpha subcomplex, 4	1.4
Ndufa5	NADH dehydrogenase (ubiquinone) 1 alpha subcomplex, 5	1.3
Ndufaf4	NADH dehydrogenase (ubiquinone) 1 alpha subcomplex, assembly factor 4	1.8
Ndufc1	predicted gene 4237; NADH dehydrogenase (ubiquinone) 1, subcomplex unknown, 1	1.6
Ndufc2	NADH dehydrogenase (ubiquinone) 1, subcomplex unknown, 2; similar to NADH dehydrogenase (ubiquinone) 1, subcomplex unknown, 2	1.3
Ndufs4	NADH dehydrogenase (ubiquinone) Fe-S protein 4	1.5
Ndufs6	similar to NADH dehydrogenase (ubiquinone) Fe-S protein 6; NADH dehydrogenase (ubiquinone) Fe-S protein 6; predicted gene 6415	1.5
Nln	neurolysin (metallopeptidase M3 family)	1.3

Nnt	nicotinamide nucleotide transhydrogenase	1.9
Nrd1	nardilysin, N-arginine dibasic convertase, NRD convertase 1	1.6
Nudt19	nudix (nucleoside diphosphate linked moiety X)-type motif 19	1.4
Ociad2	OCIA domain containing 2	2.0
Oma1	OMA1 homolog, zinc metallopeptidase (S. cerevisiae)	1.4
Opa3	optic atrophy 3 (human)	1.4
Pdha1	pyruvate dehydrogenase E1 alpha 1	1.5
Pdk1	pyruvate dehydrogenase kinase, isoenzyme 1	1.6
Pdp1	protein phosphatase 2C, magnesium dependent, catalytic subunit	2.0
Peci	peroxisomal delta3, delta2-enoyl-Coenzyme A isomerase	1.7
Perp	PERP, TP53 apoptosis effector	1.4
Phyh	phytanoyl-CoA hydroxylase	1.5
Pink1	PTEN induced putative kinase 1	1.4
Pnkd	paroxysmal nonkinesiogenic dyskinesia	1.4
Ppm1k	protein phosphatase 1K (PP2C domain containing)	1.5
Prdx6	similar to Peroxiredoxin-6 (Antioxidant protein 2) (1-Cys peroxiredoxin) (1-Cys PRX) (Acidic calcium-independent phospholipase A2) (aiPLA2) (Non-selenium glutathione peroxidase) (NSGPx); peroxiredoxin 6	1.7
Sdhaf2	RIKEN cDNA 0610038F07 gene	1.4
Sdhd	succinate dehydrogenase complex, subunit D, integral membrane protein	1.4
Sh3bp5	SH3-domain binding protein 5 (BTK-associated)	1.4
Slc25a30	solute carrier family 25, member 30	1.7
Slc25a34	solute carrier family 25, member 34	1.4
Stard7	START domain containing 7	1.4
Synj2bp	RIKEN cDNA 1810020G14 gene; synaptojanin 2 binding protein; predicted gene 4116	1.4
Tmem65	transmembrane protein 65	1.8
Tmem8b	RIKEN cDNA 4930500O05 gene	1.4
Tmtc1	transmembrane and tetratricopeptide repeat containing 1	1.5
Trmt2b	TRM2 tRNA methyltransferase 2 homolog B (S. cerevisiae)	1.4
Ttc35	tetratricopeptide repeat domain 35; predicted gene 5333	2.0
Uqcr11	ubiquinol-cytochrome c reductase (6.4kD) subunit; similar to ubiquinol-cytochrome c reductase subunit	1.5
Uqcrb	predicted gene 2962; ubiquinol-cytochrome c reductase binding protein	1.3
Uqcrh	predicted gene 14088; ubiquinol-cytochrome c reductase hinge protein	1.3
Vamp8	vesicle-associated membrane protein 8	1.6
Ywhae	tyrosine 3-monooxygenase/tryptophan 5-monooxygenase activation protein, epsilon polypeptide	1.3
0610009O20Rik	RIKEN cDNA 0610009O20 gene	1.4
1110058L19Rik	RIKEN cDNA 1110058L19 gene	1.4
2010107H07Rik	RIKEN cDNA 2010107H07 gene	1.5
9430016H08Rik	RIKEN cDNA 9430016H08 gene	2.0

Genes relate to mitochondrial (Gene Ontology cellular compartments) were compared between NTG and MCK-miR-499 gastrocnemius. 107 up-regulated genes (fold change greater than 1.3 and a significant p value < 0.05) are shown.

**Appendix Table S3. Downregulated miR-499 targets, predicted by TargetScan and MicroCosm, in MCK-miR-499 gastrocnemius muscle.**		
**Gene**	**Description**	**Fold Change**
Amd1	S-adenosylmethionine decarboxylase 1	0.47
Ankra2	ankyrin repeat, family A (RFXANK-like), 2	0.75
Ankrd27	ankyrin repeat domain 27 (VPS9 domain)	0.75
Arhgap32	Rho GTPase activating protein 32	0.83
Asb7	ankyrin repeat and SOCS box-containing 7	0.70
BC017647	family with sequence similarity 222, member B	0.71
Best3	bestrophin 3	0.74
C130073F10Rik	RIKEN cDNA C130073F10 gene	0.69
C2cd2	C2 calcium-dependent domain containing 2	0.60
Ccrn4l	nocturnin	0.79
Cdc27	cell division cycle 27	0.82
Cdkn1a	cyclin-dependent kinase inhibitor 1A (P21)	0.74
Celf2	CUGBP, Elav-like family member 2	0.79
Cgrrf1	cell growth regulator with ring finger domain 1	0.47
Cntnap2	contactin associated protein-like 2	0.64
Cpd	carboxypeptidase D	0.77
Csnk2a2	casein kinase 2, alpha prime polypeptide	0.80
Cyb5r4	cytochrome b5 reductase 4	0.69
Dcaf12	DDB1 and CUL4 associated factor 12	0.74
Dgcr8	DiGeorge syndrome critical region gene 8	0.81
Dst	dystonin	0.74
Dynlt1a	dynein light chain Tctex-type 1A	0.75
Dynlt1c	dynein light chain Tctex-type 1C	0.75
Eif4a2	eukaryotic translation initiation factor 4A2	0.79
Enpp1	ectonucleotide pyrophosphatase/phosphodiesterase 1	0.82
Fam168a	family with sequence similarity 168, member A	0.81
Fam199x	family with sequence similarity 199, X-linked	0.73
Fnip1	folliculin interacting protein 1	0.82
G6pc3	glucose 6 phosphatase, catalytic, 3	0.77
Gadl1	glutamate decarboxylase-like 1	0.49
Gm5567	transmembrane protein 178B	0.82
Gxylt1	glucoside xylosyltransferase 1	0.78
H2afz	H2A histone family, member Z	0.49
Hax1	HCLS1 associated X-1	0.68
Hgsnat	heparan-alpha-glucosaminide N-acetyltransferase	0.80
Hps5	Hermansky-Pudlak syndrome 5	0.58
Hspa1l	heat shock protein 1-like	0.78

Ide	insulin degrading enzyme	0.66
Ikzf2	IKAROS family zinc finger 2	0.74
Iscu	iron-sulfur cluster assembly enzyme	0.75
Jhdm1d	lysine (K)-specific demethylase 7A	0.81
Kcnn3	potassium intermediate/small conductance calcium-activated channel, subfamily N, member 3	0.83
Kif3c	kinesin family member 3C	0.77
Letmd1	LETM1 domain containing 1	0.80
Lrrc30	leucine rich repeat containing 30	0.73
Lysmd2	LysM, putative peptidoglycan-binding, domain containing 2	0.77
Magea5	melanoma antigen, family A, 5	0.82
Mapk6	mitogen-activated protein kinase 6	0.67
Mast4	microtubule associated serine/threonine kinase family member 4	0.62
Mdm4	transformed mouse 3T3 cell double minute 4	0.83
Mrpl37	mitochondrial ribosomal protein L37	0.69
Mtcl1	microtubule crosslinking factor 1	0.83
Mthfd1	methylenetetrahydrofolate dehydrogenase (NADP+ dependent), methenyltetrahydrofolate cyclohydrolase, formyltetrahydrofolate synthase	0.82
Mtmr2	myotubularin related protein 2	0.61
Nat1	N-acetyl transferase 1	0.74
Ndst2	N-deacetylase/N-sulfotransferase (heparan glucosaminyl) 2	0.80
Nop9	NOP9 nucleolar protein	0.76
Nr2c2	nuclear receptor subfamily 2, group C, member 2	0.77
Nup210	nucleoporin 210	0.80
Nup214	nucleoporin 214	0.80
Omg	oligodendrocyte myelin glycoprotein	0.82
Pacs2	phosphofurin acidic cluster sorting protein 2	0.71
Pam	peptidylglycine alpha-amidating monooxygenase	0.82
Pcbp2	poly(rC) binding protein 2	0.79
Pdpr	pyruvate dehydrogenase phosphatase regulatory subunit	0.68
Pfkm	phosphofructokinase, muscle	0.79
Plod3	procollagen-lysine, 2-oxoglutarate 5-dioxygenase 3	0.80
Pogz	pogo transposable element with ZNF domain	0.82
Psmd8	proteasome (prosome, macropain) 26S subunit, non-ATPase, 8	0.64
Ptar1	protein prenyltransferase alpha subunit repeat containing 1	0.67
Ptpn14	protein tyrosine phosphatase, non-receptor type 14	0.63
Ptprg	protein tyrosine phosphatase, receptor type, G	0.81
Rab5c	RAB5C, member RAS oncogene family	0.67
Ranbp2	RAN binding protein 2	0.76
Rcn2	reticulocalbin 2	0.64
Reep1	receptor accessory protein 1	0.33
Rhbdl1	rhomboid, veinlet-like 1 (Drosophila)	0.59

Rhobtb3	Rho-related BTB domain containing 3	0.62
Rnf145	ring finger protein 145	0.81
Rnf44	ring finger protein 44	0.83
Rnh1	ribonuclease/angiogenin inhibitor 1	0.69
Rpap2	RNA polymerase II associated protein 2	0.82
Rqcd1	RCD1 required for cell differentiation 1 homolog	0.77
Samd8	sterile alpha motif domain containing 8	0.77
Sdc2	syndecan 2	0.81
Sec11a	SEC11 homolog A, signal peptidase complex subunit	0.79
Sec14l1	SEC14-like lipid binding 1	0.72
Setdb1	SET domain, bifurcated 1	0.70
Sfrs9	serine/arginine-rich splicing factor 9	0.82
Slc24a2	solute carrier family 24 (sodium/potassium/calcium exchanger), member 2	0.75
Slc35a4	solute carrier family 35, member A4	0.73
Slc43a1	solute carrier family 43, member 1	0.53
Sos2	son of sevenless homolog 2 (Drosophila)	0.53
Sox6	**SRY (sex determining region Y)-box 6**	**0.65**
Spns2	spinster homolog 2	0.43
Stac3	SH3 and cysteine rich domain 3	0.68
Tbc1d22b	TBC1 domain family, member 22B	0.82
Tbrg1	transforming growth factor beta regulated gene 1	0.82
Tcf12	transcription factor 12	0.81
Tlr7	toll-like receptor 7	0.80
Tmbim6	transmembrane BAX inhibitor motif containing 6	0.64
Tmem39b	transmembrane protein 39b	0.77
Trmt44	tRNA methyltransferase 44	0.78
Trp53bp1	transformation related protein 53 binding protein 1	0.72
Tspan8	tetraspanin 8	0.23
Uap1	UDP-N-acetylglucosamine pyrophosphorylase 1	0.70
Ube2n	ubiquitin-conjugating enzyme E2N	0.82
Ubn1	ubinuclein 1	0.77
Ubn2	ubinuclein 2	0.77
Uevld	UEV and lactate/malate dehyrogenase domains	0.63
Usp12	ubiquitin specific peptidase 12	0.75
Vps53	VPS53 GARP complex subunit	0.79
Wdr35	WD repeat domain 35	0.72
Zbtb22	zinc finger and BTB domain containing 22	0.79
Zc3hav1	zinc finger CCCH type, antiviral 1	0.83
Zdbf2	zinc finger, DBF-type containing 2	0.77
Zfp26	zinc finger protein 26	0.74
Zfp266	zinc finger protein 266	0.78

Zfp42	zinc finger protein 42	0.79
Zscan4c	zinc finger and SCAN domain containing 4C	0.79

mRNA expression was assessed on and analyzed using Partek Genomics Suite. Downregulation was defined as a fold-change <-1.2.

Appendix Table S4. RT-PCR primers		
**Mouse Gene**	**Forward**	**Reverse**
36b4	5'-ATCCCTGACGCACCGCCGTGA	5'-TGCATCTGCTTGGAGCCCACGT
Ndufa1	5'-TGA TGG AAC GCG ATA GAC G	5'-GCC AGG AAA ATG CTT CCT TA
ndufs4	5'-GAT GGG AAA ATC CTT TGA TGG	5'-GAA GGT CAG AAC CAT GTT GGA
Ndufaf4	5'-AAA ATG AGA GCT GAG AGA GCT GTT	5'-ACT TTA CCC AAA CCA CCC ACT
Sdhd	5'-CCT GCT CTG TGG TGG ACT ACT	5'-CCC ATG AAC GTA GTC GGT AAC
Uqcr11	5'-CCACAGGCCTCGATGGTA	5'-GCAGCCCTAGTGTCTGTCAA
Cox7a1	5'-CGA AGA GGG GAG GTG ACT C	5'-AGC CTG GGA GAC CCG TAG
Cox7b	5'-AAC GCA CTA AGC CGT CTC C	5'-CAT GGA AAC TAG GTG CCC TCT
Atp5e	5'-CAA AGC GAA CGC TGA GAA G	5'-TCA GCA CTT CAG GCT TCA GA
Atp5l	5'-AAA ACT GGT AGC TTC AAA CAC CTT	5'-ACA ATG CCA CGT TTG CCT AT
mt-Nd2	5'-CCATCAACTCAATCTCACTTCTATG	5'-GAATCCTGTTAGTGGTGGAAGG
mt-Co1	5'-ACCATCATTTCTCCTTCTCCTA	5'-TAGATTTCCGGCTAGAGGTG
mt-Co2	5'-TGAAGACGTCCTCCACTCATG	5'-CCCTGGTCGGTTTGATGTTA
mt-Atp6	5'-CCATAAATCTAAGTATAGCCATTCCAC	5'-AGCTTTTTAGTTTGTGTCGGAAG
mt-Cytb	5'-CATTTATTATCGCGGCCCTA	5'-TGGGTTGTTTGATCCTGTTTC
Cycs	5'-ACCAAATCTCCACGGTCTGTT	5'-GGATTCTCCAAATACTCCATCAG
Lpl	5'-TTTGTGAAATGCCATGACAAG	5'-CAGATGCTTTCTTCTCTTGTTTGT
Ldha	5'-TGCCTACGAGGTGATCAAGCT	5'-GCACCCGCCTAAGGTTCTTC
Ldhb	5'-AGTCTCCCGTGCATCCTCAA	5'-AGGGTGTCCGCACTCTTCCT
Ppargc1a	5'-CGGAAATCATATCCAACCAG	5'-TGAGAACCGCTAGCAAGTTTG
Ppargc1b	5'-TCCAGAAGTCAGCGGCCT	5'-CTGAGCCCGCAGTGTGG
Esrra	5'-AGGAGTACGTCCTGCTG	5'-CCTCAGCATCTTCAATG
Esrrb	5'-ACGGCTGGATTCGGAGAAC	5'-TCCTGCTCAACCCCTAGTAGATTC
Esrrg	5'-TGACTTGGCTGACCGAG	5'-CCGAGGATCAGAATCTCC
Ppara	5'-ACTACGGAGTTCACGCATGTG	5'-TTGTCGTACACCAGCTTCAGC
Ppard	5'-TCACCGGCAAGTCCAGCCA	5'-ACACCAGGCCCTTCTCTGCCT
Myh7	5'-GCCAACTATGCTGGAGCTGATGCCC	5'-GGTGCGTGGAGCGCAAGTTTGTCATAAG
Myh7b	5'-GCCTCTGCGGACATTGATAG	5'-GGGCAGCTGGAAGATCACT
Tnni1	5'-TGAAGCCAAATGCCTCCACAACAC	5'-ACACCTTGTGCTTAGAGCCCAGTA
Tnnc1	5'-AGCTCATGAAGGACGGTGACAAGA	5'-AACCGTGCAAGACCAGCATCTACT
Tnnt1	5'-TGGATCCACCAGCTGGAATCAGAA	5'-GCTGATGCGGTTGTAGAGCACATT
Tnni2	5'-AGCAGCAAGGAGCTGGAAGA	5'-ATGGCGTCGGCAGACATAC
Tnnc2	5'-CCATCATCGAGGAGGTGGAC	5'-CTTCCCCTTCGCATCCTCTT
Tnnt3	5'-AACTGGAGACTGACAAATTCGAGT	5'-GCTGTGCTTCTGGGTTTGGT
Sox6	5'- TCTACAGCAGCAGCACAAGATTA	5'- CATGTGGAAAAATTGGGATCA
Fnip1	5'- TCCGTCAGTGCCTGGTATC	5'- ACAGCTTCTGCTATTGGTTCATC
Fnip2	5'- TTGACTCCAGAGCCGTTCA	5'- GGAACATCCTCTGCTTTCTGA
**Primers for quantification of mtDNA**		
mt-Nd1	5'-CCCATTCGCGTTATCTT	5'-AAGTTGATCGTAACGGAAGC
Lpl	5'-GATGGACGGTAAGAGTGATTC	5'-ATCCAAGGGTAGCAGACAGGT
