# Supplementary material for: Coupling of mitochondrial function and skeletal muscle fiber type by a miR‐499/Fnip1/AMPK circuit
Source: EMBO Mol Med. 2016 Aug 9;8(10):1212–28. doi: 10.15252/emmm.201606372 (PMC5048369; doi:10.15252/emmm.201606372)
Supplement: Supplementary file 2 — Expanded View Figures PDF [file EMMM-8-1212-s002.pdf]

Expanded View Figures

A

Upstream Regulator	Fold Change	Predicted Activation State	Activation z-score
Ppargc1a	2.191	Activated	2.804
Cd38	1.498	Activated	3.060
Tlr3	1.371	Activated	3.274
Mapkapk2	1.352	Activated	2.236
Irf7	1.337	Activated	2.588

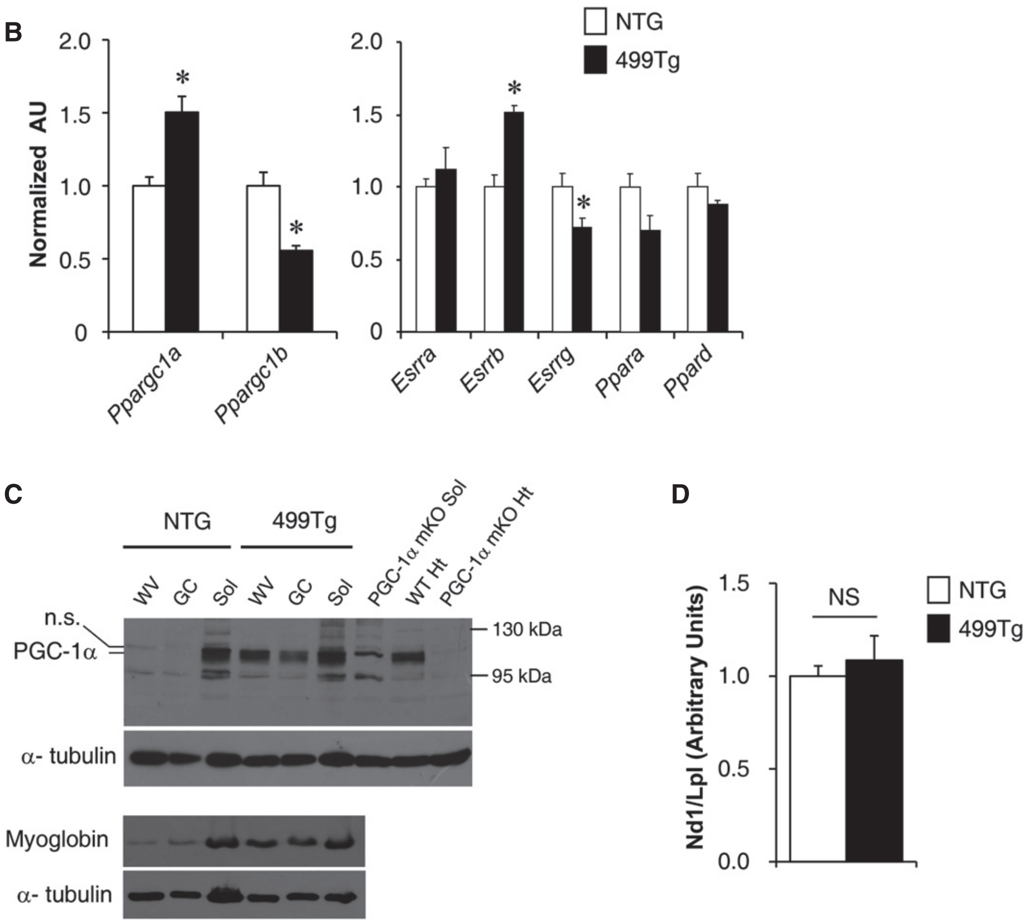

Figure EV1. Increased PGC-1α expression in MCK-miR-499 muscle.

A Upstream regulator analysis by Ingenuity Pathways Analysis (IPA) based on the gene expression array data generated from the gastrocnemius muscle of MCK-miR-499. The top five upstream regulators are shown with the predicted z-scores.

B Expression of genes encoding PGC-1, ERR, and PPAR transcription factors (RT-qPCR) in gastrocnemius muscle compared to NTG controls (n = 5 mice per group). *$P < 0.05$.

C Representative Western blot analysis of PGC-1α (Top) and myoglobin (Bottom) in white vastus (WV), gastrocnemius (GC), and soleus (Sol) muscle from the indicated genotypes (n = 4 mice per group).

D Results of qPCR to determine mitochondrial DNA levels in WV muscle of the indicated genotypes using primers for NADH dehydrogenase (*Nd1*, mitochondria-encoded) and lipoprotein lipase (*Lpl*, nuclear-encoded). *Nd1* levels were normalized to *Lpl* DNA content and expressed relative to NTG (= 1.0) muscle (n = 5 mice per group). P = 0.569 (NS, not significant).

Data information: All values represent the mean ± SEM. P-value was determined using two-tailed unpaired Student's t-test.
Source data are available online for this figure.

 © 2016 The Authors

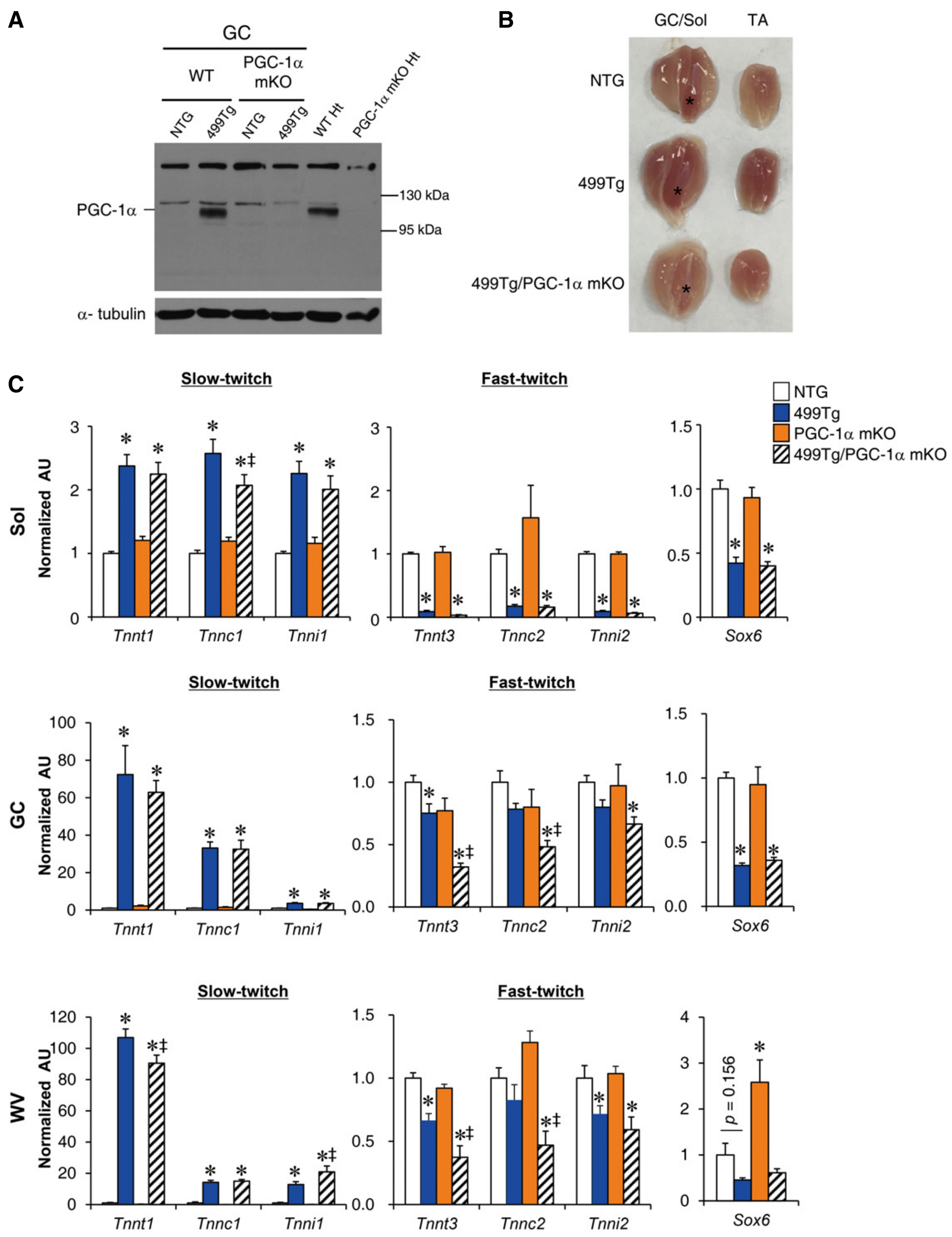

Figure EV2. PGC-1α-independent regulation of the slow-twitch muscle fiber program by miR-499.

A Representative Western blot analysis of PGC-1α protein expression in the gastrocnemius muscle from the indicated genotypes (n = 4 mice per group).

B Morphology of dissected gastrocnemius/soleus (asterisk) and tibialis anterior (TA) muscle.

C Expression of genes encoding slow-twitch/fast-twitch troponin and *Sox6* (RT-qPCR) in soleus (Sol), gastrocnemius (GC), and white vastus (WV) muscle from the indicated genotypes. For Sol, NTG, n = 10; 499Tg, n = 9; PGC-1α mKO, n = 8; 499Tg/PGC-1α mKO, n = 10. For GC and WV, n = 5 mice per group. $^{*}P < 0.05$ (versus NTG), $^{‡}P < 0.05$ (versus 499Tg).

Data information: Values represent the mean (± SEM) and are shown as arbitrary units (AU) normalized (= 1.0) to the value of the NTG control. *P*-value was determined using one-way ANOVA coupled to a Fisher's least-significant difference (LSD) *post hoc* test.
Source data are available online for this figure.

© 2016 The Authors

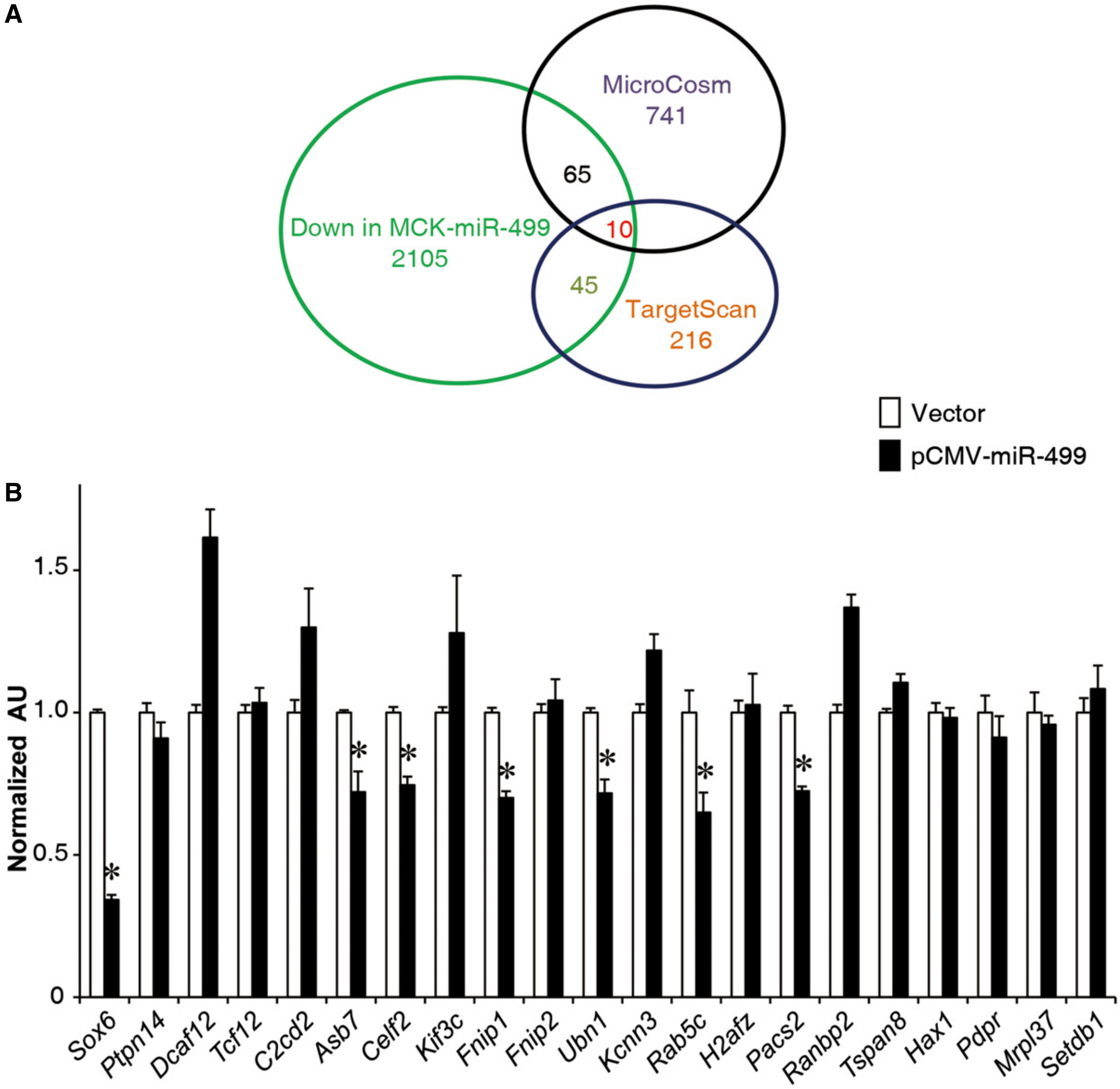

Figure EV3. Identification of miR-499 targets.

A Diagram shows the miR-499 target identification. The TargetScan and MicroCosm programs were used to identify putative target mRNAs for miR-499, and this list was cross-matched for genes that were downregulated in MCK-miR-499 muscle (fold change < −1.2). The overlapping putative targets, together with those predicted targets that are known to be involved in the regulation of energy metabolism, were chosen for further UTR luciferase validation assay.

B 3′UTR luciferase reporters containing the predicted binding site of miR-499 were used in cotransfection studies in HEK293T cells in the presence or absence of plasmids expressing miR-499 (n = 3 independent experiments). *Sox6* 3′UTR containing the binding site of miR-499 was used as a positive control. *$P < 0.01$. All values represent the mean ± SEM and are shown as arbitrary units (AU) normalized to corresponding controls. P-value was determined using two-tailed unpaired Student's t-test.

 © 2016 The Authors

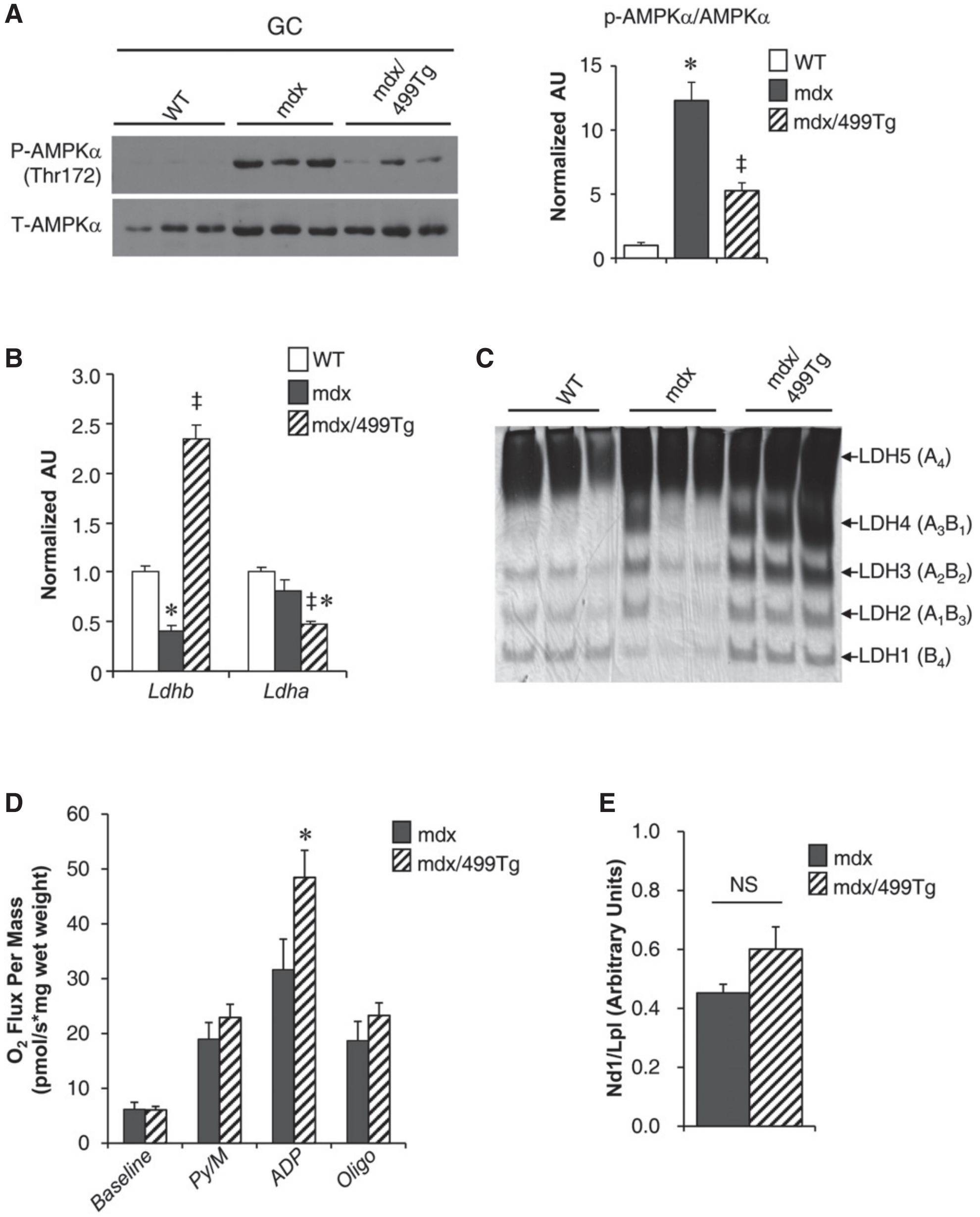

Figure EV4. miR-499 activation reverses the diminished oxidative muscle fiber program in mdx muscle.

A (Left) Representative Western blot analysis performed on extracts of the gastrocnemius muscle isolated from the indicated genotypes using phospho-AMPKα (Thr172) and AMPKα antibodies. (Right) Quantification of the p-AMPKα/AMPKα signal ratios normalized (= 1.0) to the NTG control. WT, n = 6; mdx, n = 6; mdx/499Tg, n = 5. $^*P < 0.0001$ (versus WT), $^‡P < 0.0001$ (versus mdx).

B Expression of the *Ldhb* and *Ldha* genes (RT-qPCR) in muscle from the indicated genotypes (n = 5 mice per group). *Ldhb*: $^*P = 0.0006$ (versus WT), $^‡P < 0.0001$ (versus mdx); *Ldha*: $^*P = 0.059$ (versus WT), $^‡P = 0.0052$ (versus mdx).

C A representative LDH isoenzyme activity gel is shown (n = 3 mice per group).

D Mitochondrial respiration rates were determined from the extensor digital longus muscle of the indicated genotypes using pyruvate/malate as substrate. Pyruvate/malate (Py/M)-stimulated, ADP-dependent respiration, and oligomycin-induced (oligo) are shown. mdx, n = 6; mdx/499Tg, n = 7. $^*P = 0.0474$ (ADP).

E Results of qPCR to determine mitochondrial DNA levels in WV muscle of the indicated genotypes using primers for NADH dehydrogenase (*Nd1*, mitochondria-encoded) and lipoprotein lipase (*Lpl*, nuclear-encoded). mdx, n = 5; mdx/499Tg, n = 6. $P = 0.0989$ (NS, not significant).

Data information: All values represent the mean ± SEM. *P*-value in (A and B) was determined using one-way ANOVA coupled to a Fisher's least-significant difference (LSD) *post hoc* test; *P*-value in (D and E) was determined using two-tailed unpaired Student's *t*-test.
Source data are available online for this figure.

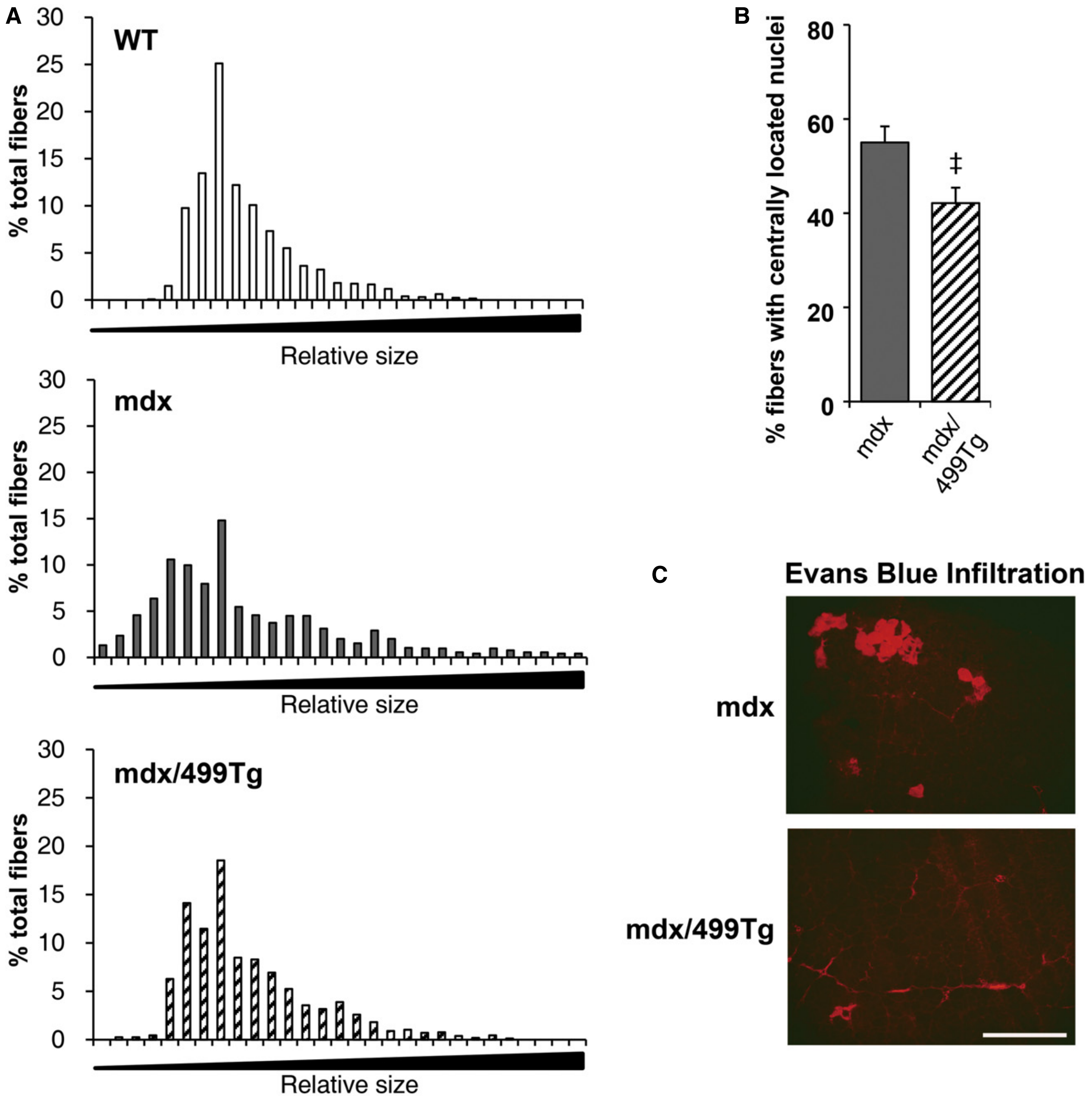

Figure EV5. Restoring the expression of miR-499 reduces muscle damage in mdx mice.

A Bar histograms represent size distribution of the muscle fibers from the indicated genotypes. Relative fiber size was quantified using Image-Pro Plus software (n = 5 mice per group).

B Quantification of muscle fibers with centrally located nuclei in indicated genotypes. Values represent the mean % (± SEM) total muscle fibers from n = 5 mice per group and five images per muscle. $^*P$ = 0.0306 (versus mdx).

C Representative images of Evans blue dye infiltration in damaged myofibers of the tibialis anterior (TA) muscle from indicated genotypes. Scale bar: 500 µm.

Data information: Values represent the mean ± SEM. P-value was determined using two-tailed unpaired Student's t-test.

 © 2016 The Authors
