# Supplementary figures and images for: Coupling of mitochondrial function and skeletal muscle fiber type by a miR‐499/Fnip1/AMPK circuit
Source: EMBO Mol Med. 2016 Aug 9;8(10):1212–28. doi: 10.15252/emmm.201606372 (PMC5048369; doi:10.15252/emmm.201606372)

Fig EV1C

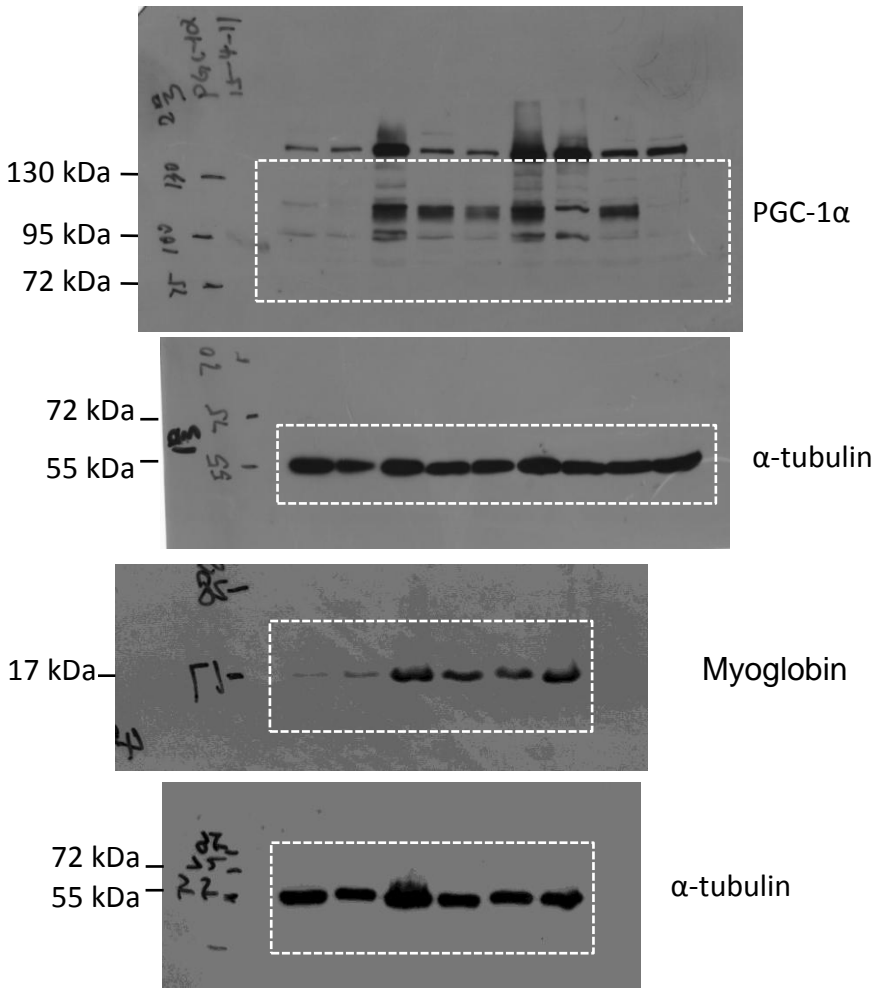

Supplement: Supplementary file 3 — Source Data for Expanded View [file EMMM-8-1212-s008.zip › Source_Data_for_Expanded_View/SourceDataForExpandedView1.pdf]

Fig EV2A

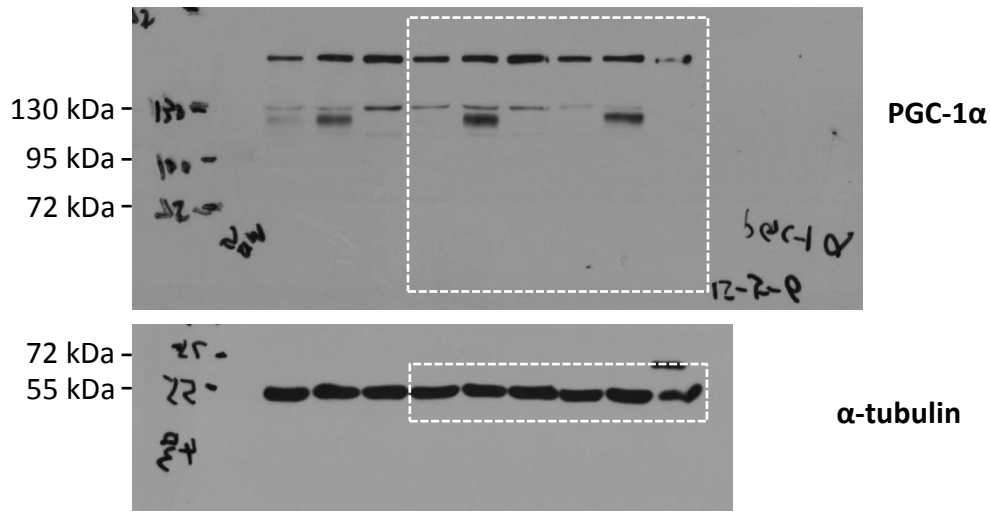

Supplement: Supplementary file 3 — Source Data for Expanded View [file EMMM-8-1212-s008.zip › Source_Data_for_Expanded_View/SourceDataForExpandedView2.pdf]

SourceDataForExpandedView4. Original uncropped gels

Fig EV4A

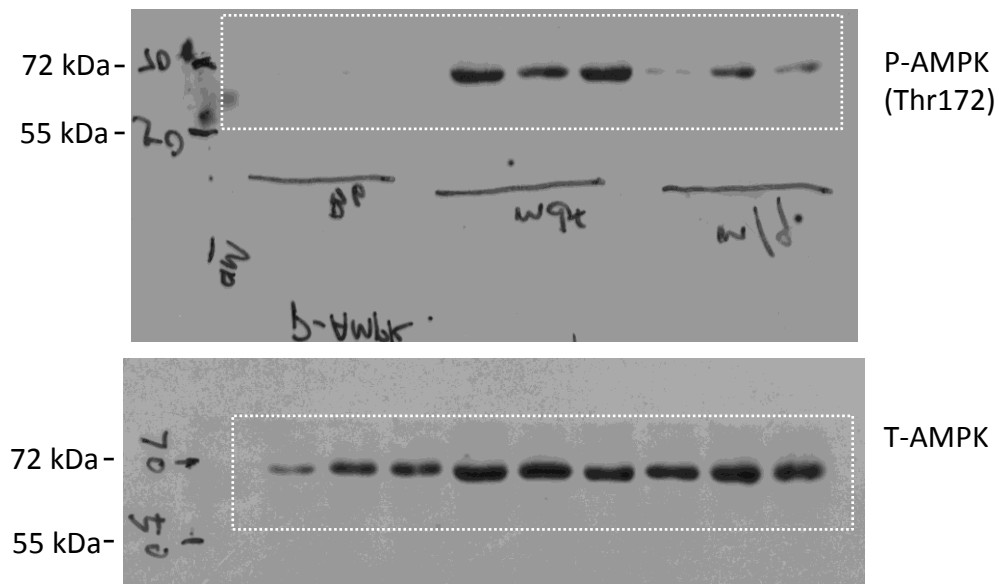

Fig EV4C

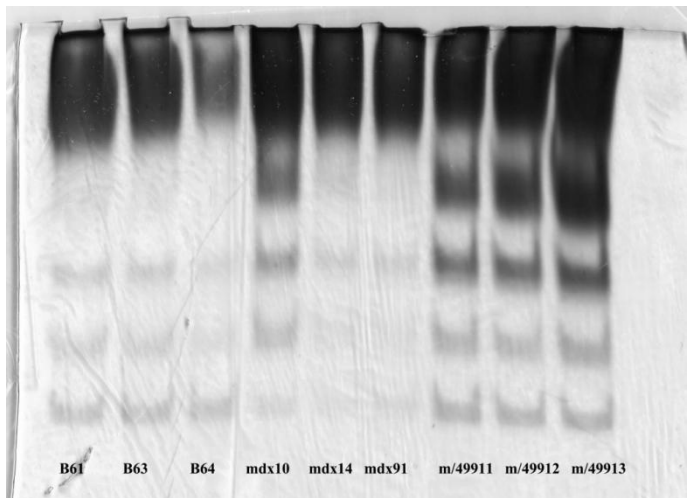

Supplement: Supplementary file 3 — Source Data for Expanded View [file EMMM-8-1212-s008.zip › Source_Data_for_Expanded_View/SourceDataForExpandedView4.pdf]

SourceDataForFigure2. Original uncropped gels

Fig 2C

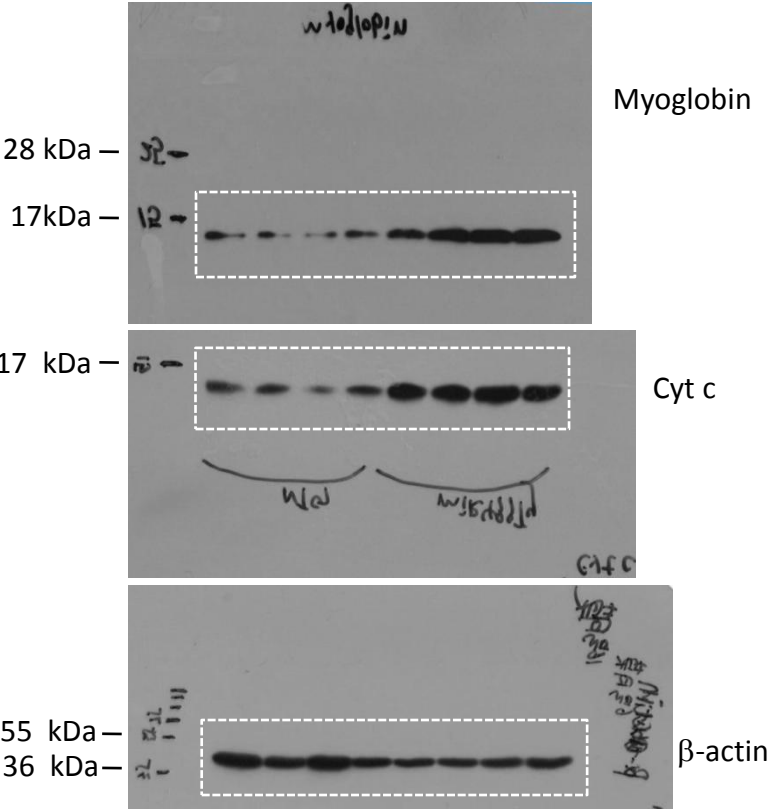

Fig 2E

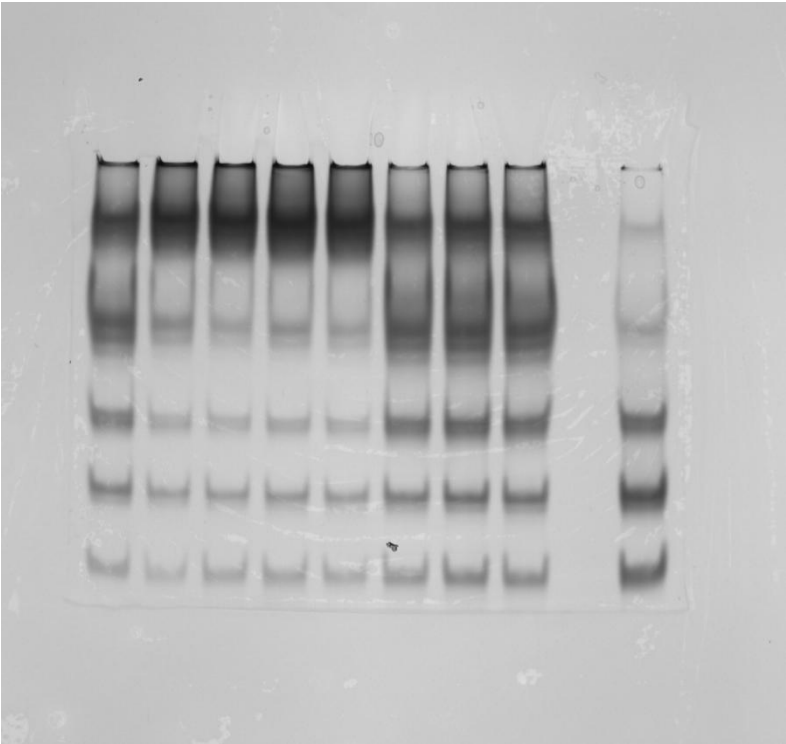

Supplement: Supplementary file 5 — Source Data for Figure 2 [file EMMM-8-1212-s003.pdf]

SourceDataForFigure3. Original uncropped gels

Fig 3A

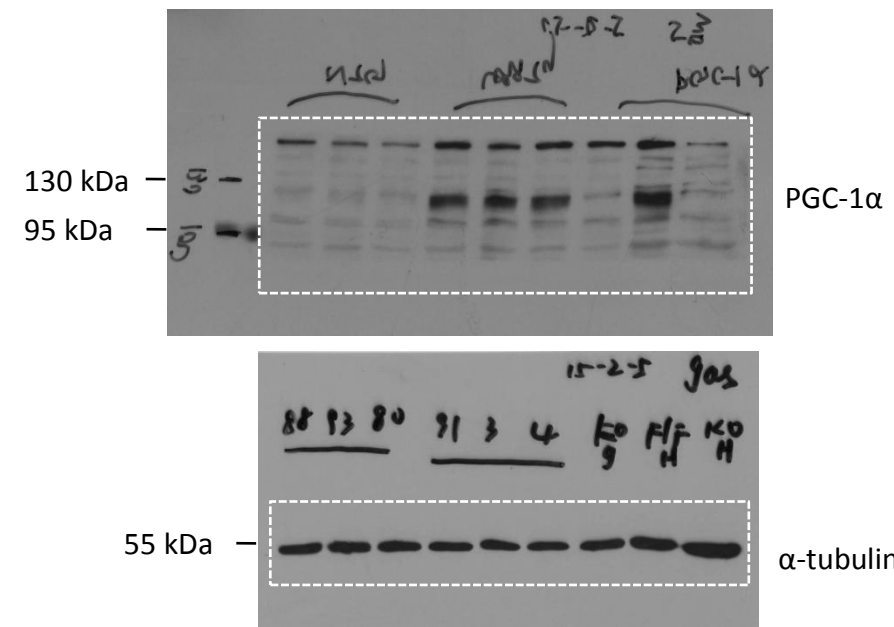

Fig 3C

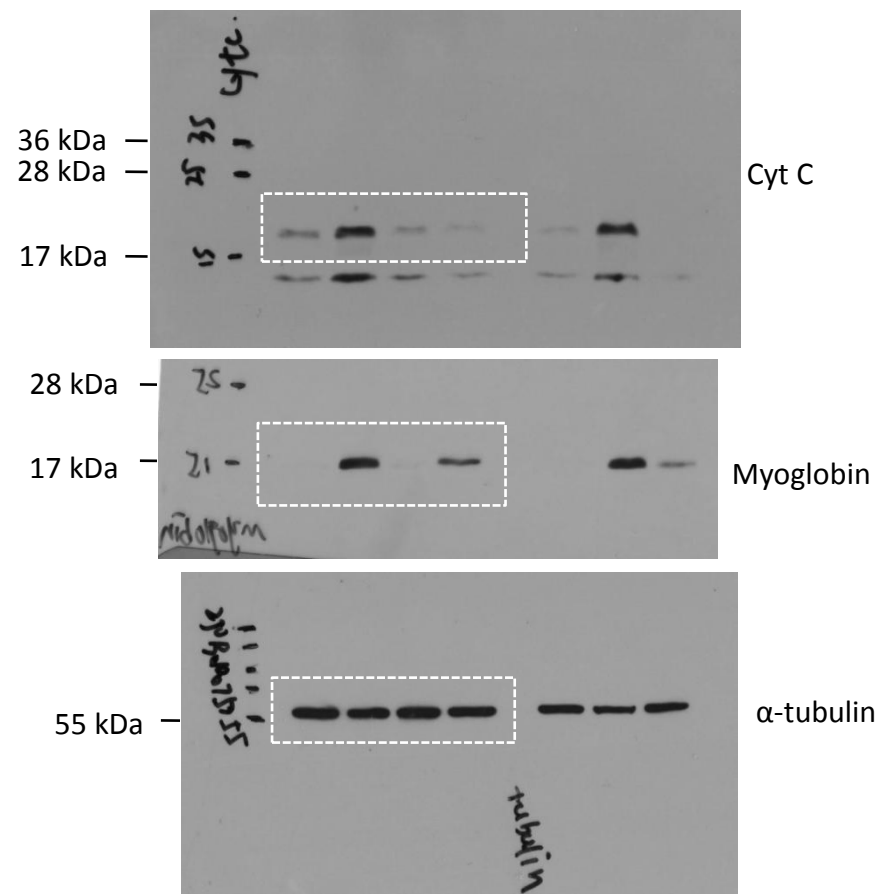

Supplement: Supplementary file 6 — Source Data for Figure 3 [file EMMM-8-1212-s004.pdf]

SourceDataForFigure4. Original uncropped gels

Fig 4D

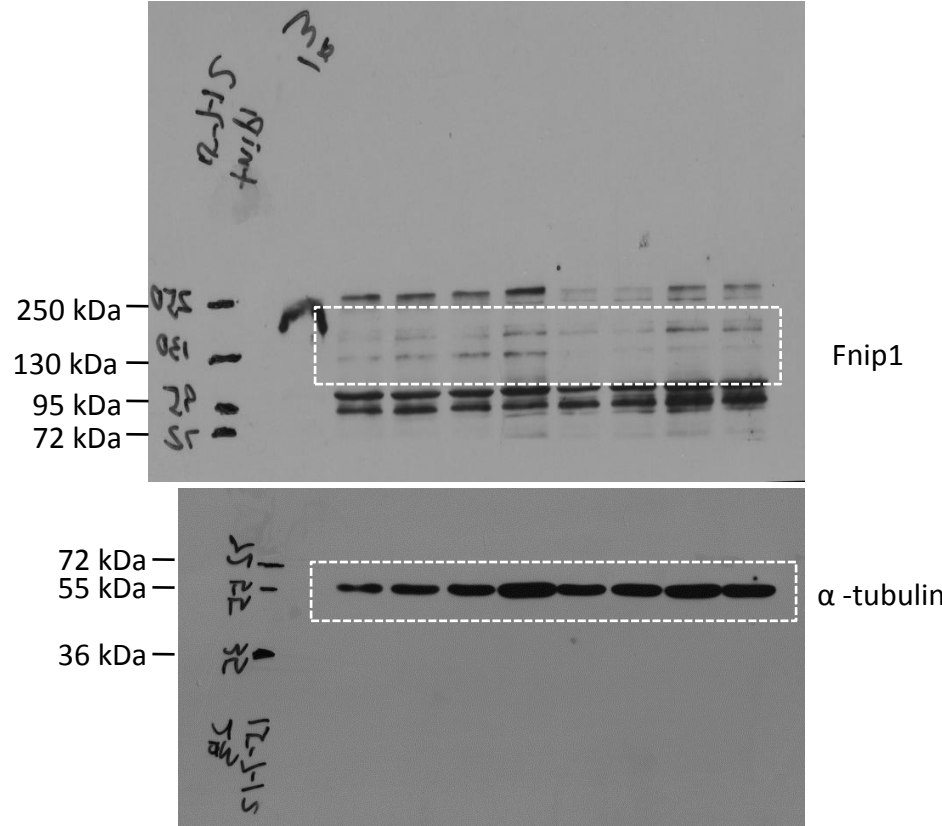

Supplement: Supplementary file 7 — Source Data for Figure 4 [file EMMM-8-1212-s005.pdf]

SourceDataForFigure5. Original uncropped gels

Fig 5A

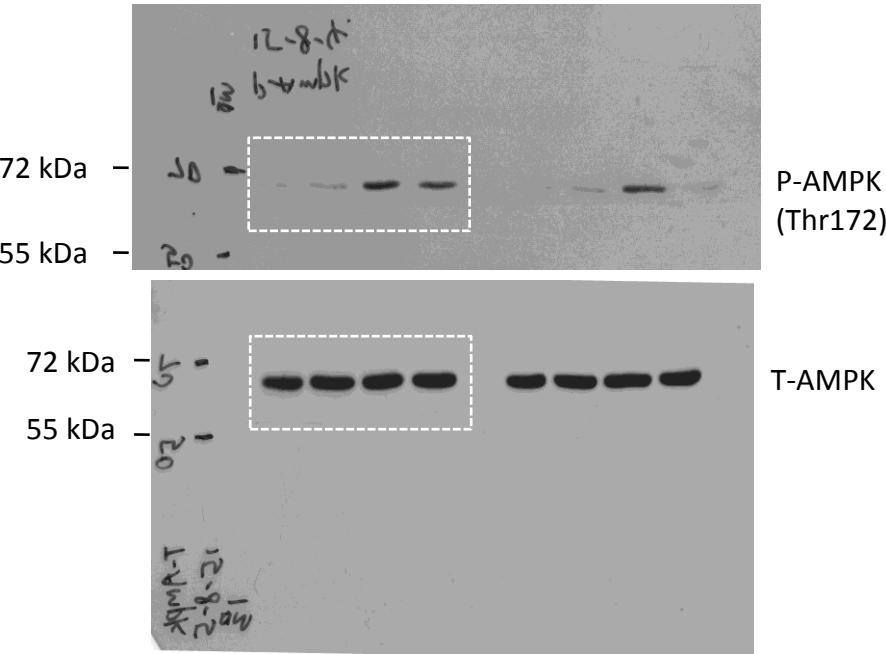

Fig 5D

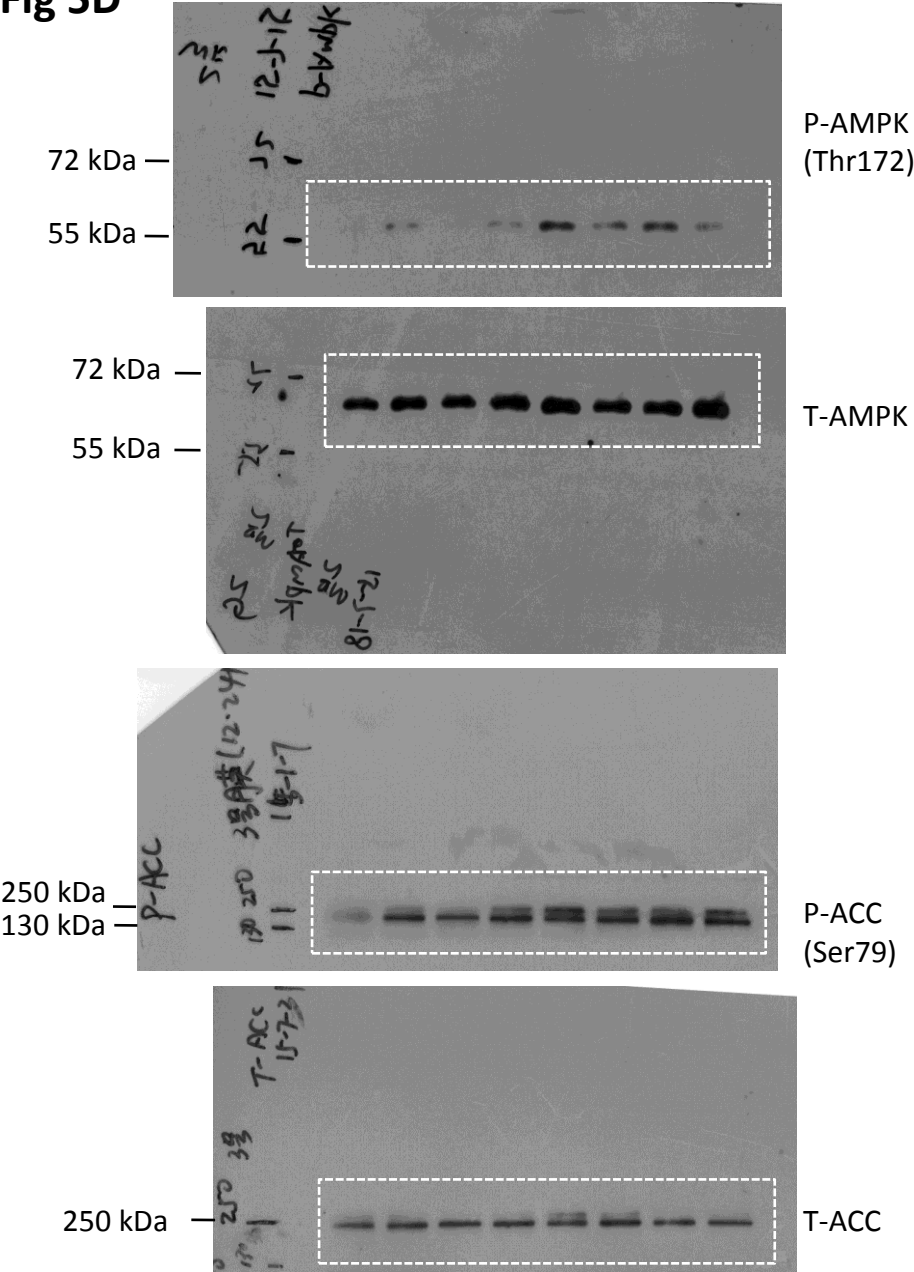

Supplement: Supplementary file 8 — Source Data for Figure 5 [file EMMM-8-1212-s006.pdf]

SourceDataForFigure6. Original uncropped gels

Fig 6C

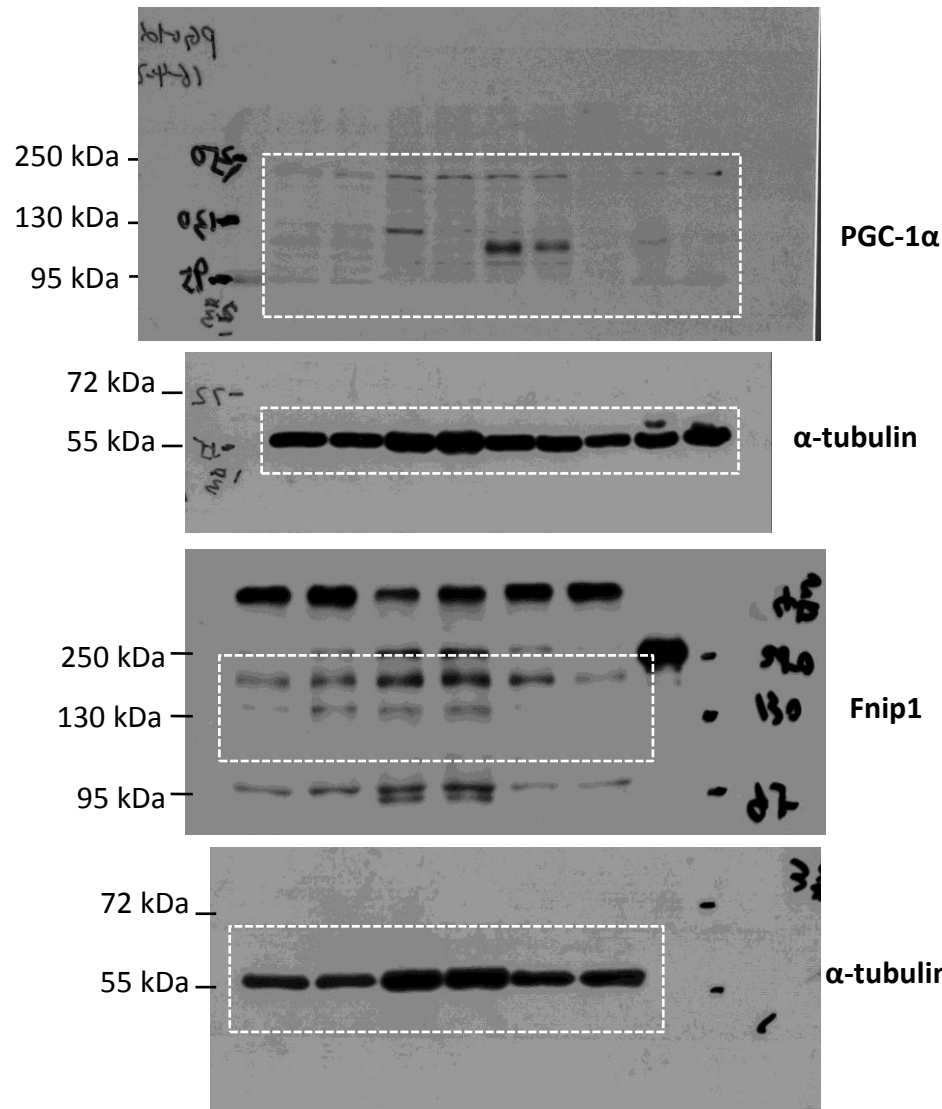

Fig 6D

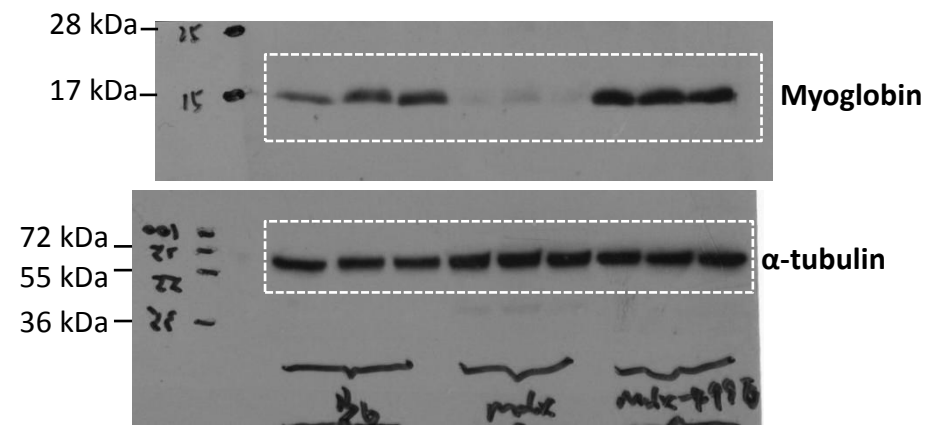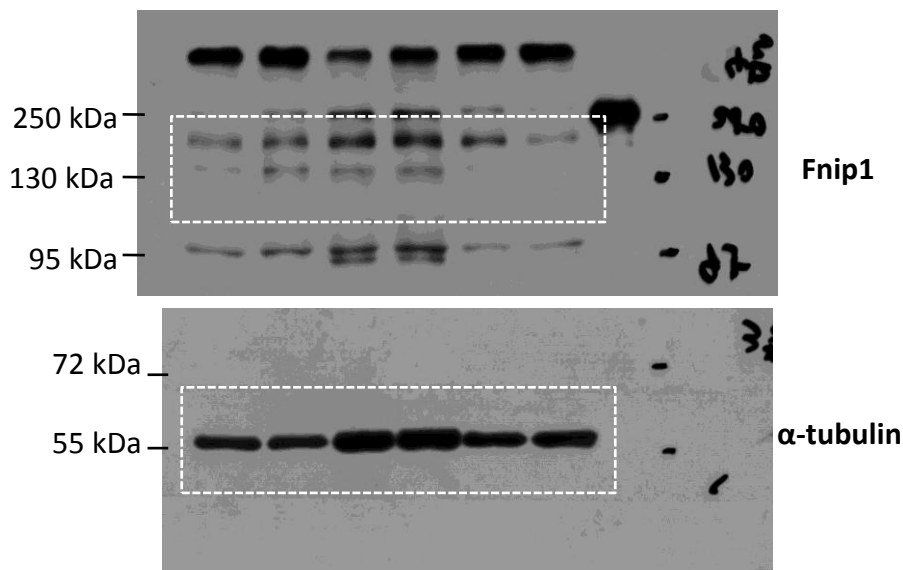

Supplement: Supplementary file 9 — Source Data for Figure 6 [file EMMM-8-1212-s007.pdf]
